# Effects of Melatonin on Diabetic Neuropathy and Retinopathy

**DOI:** 10.3390/ijms23010100

**Published:** 2021-12-22

**Authors:** Klausen Oliveira-Abreu, José Cipolla-Neto, Jose Henrique Leal-Cardoso

**Affiliations:** 1 Laboratório de Eletrofisiologia, Instituto Superior de Ciências Biomédicas, Universidade Estadual do Ceará, Fortaleza 60714-903, CE, Brazil; klausenoliveira@gmail.com; 2 Laboratório de Neurobiologia, Instituto de Ciências Biomédicas 1, Universidade de São Paulo, Sao Paulo 05508-000, SP, Brazil; cipolla@icb.usp.br

**Keywords:** melatonin, diabetes mellitus, diabetic neuropathy, diabetic retinopathy

## Abstract

Diabetes mellitus (DM) leads to complications, the majority of which are nephropathy, retinopathy, and neuropathy. Redox imbalance and inflammation are important components of the pathophysiology of these complications. Many studies have been conducted to find a specific treatment for these neural complications, and some of them have investigated the therapeutic potential of melatonin (MEL), an anti-inflammatory agent and powerful antioxidant. In the present article, we review studies published over the past 21 years on the therapeutic efficacy of MEL in the treatment of DM-induced neural complications. Reports suggest that there is a real prospect of using MEL as an adjuvant treatment for hypoglycemic agents. However, analysis shows that there is a wide range of approaches regarding the doses used, duration of treatment, and treatment times in relation to the temporal course of DM. This wide range hinders an objective analysis of advances and prospective vision of the paths to be followed for the unequivocal establishment of parameters to be used in an eventual therapeutic validation of MEL in neural complications of DM.

## 1. Introduction

Diabetes mellitus (DM) is a metabolic disorder characterized by hyperglycemia due to chronic or relative insulin deficiency. DM is associated with a high mortality rate, complications, and various comorbidities. The main complications of DM are nephropathy, retinopathy, and neuropathy [1,2].

Diabetic neuropathy (DN) affects more than 50% of patients with diabetes and is defined as a set of clinical syndromes that affect different regions of the nervous system [3]. Diabetic retinopathy (DR) is often asymptomatic and one of the main causes of vision loss worldwide [4]. There are few treatments available for the treatment of DN and DR, and most of them are ineffective and have serious side effects; therefore, an alternative or complementary treatment is urgently sought. The compound melatonin (MEL) has been studied for the treatment of DM and its complications, such as DN and DR [4].

MEL is a hormone produced by the pineal gland. The physiological plasma concentration of MEL varies during the day, reaching its peak at night, and can vary according to other factors, mainly ambient light level, age, sex, tissue of collection and etc. According to different reports, the peak of the nocturnal plasma concentration of MEL is in the picomolar range [5,6,7,8,9].

MEL has several pharmacological effects, including neuroprotective, anti-inflammatory, and inhibitory actions on the excitability of neurons in the central nervous system (CNS) and peripheral nervous system (PNS) [8,10,11]. Additionally, MEL has high pharmacological potency (a significant effect is observed from a 100 pM concentration [12]) and a powerful antioxidant effect, suggesting that it may be effective in the treatment of DN [8,10].

Furthermore, MEL has a low toxicity, and most experimental tests have used MEL in a concentration of 0.5–5 mg/kg. However, even at a dose of 800 mg/kg in rats, MEL did not cause any measurable toxic adverse effects. The LD_50_ for intraperitoneal injection has been reported to be 1168 mg/kg in rats and 1131 mg/kg in mice [13].

The aim of this article was to review previous studies reporting the effects of MEL on DN and DR and assess the extension and magnitude of the subsidies they provide for the possible therapeutic use of MEL for the treatment of DM and its complications.

## 2. Diabetic Neuropathy and Retinopathy

### 2.1. General Aspects of Epidemiology and Physiopathology of Diabetic Neuropathy and Retinopathy

DN is one of the most prevalent complications of DM and is characterized by a clinical syndrome mainly resulting from damage to the peripheral and autonomic nervous systems [3].

DN is a common complication in both type 1 and type 2 DM. Additionally, several studies have suggested that pre-diabetic patients present with some types of neuropathy. DN has a considerable impact on the patient’s quality of life and can cause several complications, including weakness, ataxia, and lack of coordination, all of which predispose patients to falls and fractures [14]. DN can be classified as diffuse or focal neuropathy. Diffuse neuropathies are common, usually chronic, and often progressive. Focal neuropathies are less common, usually of acute onset, and often self-limited [15].

The pathophysiology of DN is not fully understood but is likely to be multifactorial [16]. Oxidative and nitrosative stress, accumulation of glycation end products, microvascular insufficiency, derangements in normal metabolic homeostasis, and persistent hyperglycemia are some of the factors postulated to be involved in the pathogenesis of DN [14,16]. DR is one of the most serious and common microvascular complications of DM and is the leading cause of blindness worldwide. Despite recent advances, the pathophysiological mechanisms of DR remain poorly understood. A mechanism proposed to be related to DR is the increase in oxidative stress observed in DM.

Similarly to DN, the mechanisms involved in the pathogenesis of DR are not well understood. However, recent studies have associated biochemical mechanisms with DR, such as oxidative stress, activation of protein kinase C isoforms, polyol and hexosamine pathway activity, and inflammation [17,18].

Owing to its epidemiological importance, several models seek to reproduce the main characteristics of DM in experimental animals [19,20,21]. One of the most commonly used models in the scientific literature is DM induction through an injection of streptozotocin (STZ). STZ is an antibiotic produced by *Streptomyces achromogenes* that selectively destroys pancreatic β cells, thus interfering with insulin synthesis and consequently, glycemic control [19,20]. Using STZ as a model, previous studies have reported important changes caused by DM in the CNS [22] and PNS, such as in the dorsal root ganglia (DRG) [23] and in the superior cervical ganglion (SCG) of Wistar rats [24].

Noteworthy electrophysiological effects caused by DM on PNS neurons include increased rheobase and chronaxie of the compound action potential of the sciatic nerve of rats induced to DM on the 5th day of life (n5-STZ), suggesting an alteration of excitability. Regarding the intracellular recorded action potential (AP) of isolated DRG neurons, a statistically significant depolarization of the resting membrane potential (RMP) and a tendency towards a reduction in the rheobase have been observed in n5-STZ model [25].

The SCG, in turn, is one of the possible targets of the autonomic dysfunction caused by DM and is also responsible for the innervation of the pineal gland through postganglionic sympathetic fibers, thus influencing the synthesis and secretion of MEL [26]. The effect of DM-induced alterations on the SCG in adult animals has been characterized. One month after STZ-induced diabetes, RMP depolarization was reported in the most frequent type of neurons found in the SCG, known as single firing phasic (SFP) neurons. Furthermore, in the same paper, a reduction in input resistance (R_in_) was observed in other neuronal types, known as multiple firing phasic neurons, suggesting that the effects of DM on the observed passive membrane properties are widespread, but dependent on neuronal type. Regarding the active electrophysiological properties, a tendency for rheobase reduction was observed, suggesting increased excitability of SFP neurons. Finally, although the effects of DM were observed for a relatively short period, they were sufficient to inhibit Na^+^K^+^ATPAse pump activity [24].

### 2.2. Relationship of Melatonin with Diabetic Neuropathy and Retinopathy

Several studies have proposed an interrelationship between MEL and DM. Some studies have suggested that MEL can be used to treat DM and its complications, such as DN and DR, while others report that DM can cause changes in the pineal gland or MEL synthesis.

#### 2.2.1. Effect of Diabetes Mellitus on the Pineal Gland

Studies on the effect of DM on the pineal gland have reported a progressive reduction in the total area of the pineal gland and the nuclear diameter of pinealocytes in the 4th week after DM induction [26]. Furthermore, in nerve fibers containing the 9.5 protein gene product, a general marker for nerve fibers, the presence of this marker was significantly attenuated in the pineal of diabetic animals, suggesting the development of neuropathy. Six months after the development of DM, alteration of glial cells through thickening of the axon-Schwann cell units was also reported. Despite these findings, there was no evidence of cell death during the study period.

The effects of DM on the pineal gland were also analyzed using Goto–Kakizaki (GK) diabetic rats and male Wistar rats as controls [27]. GK rats are a non-obese strain that has been established as a polygenic model of type 2 DM (T2DM). The pineal gland of diabetic rats presented lower weight and a lower amount of norepinephrine in the 50th week of life. Moreover, regarding the amount of protein and response to noradrenaline, a reduction was observed at eight and 50 weeks of life in diabetic animals compared to controls. The pineal gland reaction in diabetic rats was also reduced following stimulation with norepinephrine. These data led the authors to suggest that DM may cause hypotrophy of the pineal gland, possibly due to reduced glucose uptake [27].

**Figure 1:**
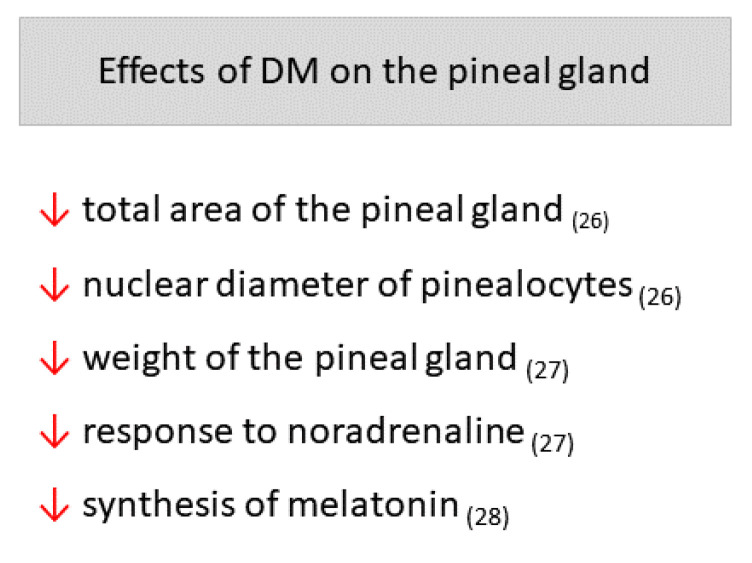
Main effects of Diabetes mellitus (DM) on the pineal gland. Arrows point down: decrease. The respective references can be found subscribed in parentheses.

Amaral et al. [28] measured the synthesis of MEL using microdialysis of the pineal gland, which enabled continuous monitoring of the same animal before and after DM induction by STZ. An acute and sustained reduction in MEL synthesis in Wistar rats was reported soon after DM induction. According to the authors, this reduction was not related to pinealocyte necrosis or apoptosis; it was suggested to be a direct consequence of the hyperglycemic state affecting pineal MEL production through reduction in noradrenergic signaling and Na^+^K^+^ATPase activity. The main effects of DM on the pineal gland can be found in Figure 1.

#### 2.2.2. Effect of Melatonin Treatment on Diabetic Neuropathy in Experimental Studies

Several studies have reported the effects of MEL treatment on DM and DN using different approaches. As DM increases the formation of oxygen and nitrogen free radicals in the body, one of the most researched approaches is the possible antioxidant effect of MEL on DM animal models. It has been reported that DM causes an increase in oxidative stress by measuring different markers, such as increased malondialdehyde (MDA), lipid peroxidation, and reduced levels of glutathione-S-transferase (GSH), superoxide dismutase (SOD), and catalase. MEL has been shown to reverse the change in the levels of all of these markers, in addition to increasing the total antioxidant capacity (TAC) [29,30,31,32,33].

In addition, as DM is considered an inflammatory disease, some studies have measured the effect of DM and MEL treatment on pro-inflammatory cytokines. DM has been shown to increase the levels of Tumor Necrosis Factor alpha (TNF-α), interleukin 6 (IL-6), inducible nitric oxide synthase (iNOS), and the adipokine leptin, while treatment with MEL reverted these parameters to normal values, suggesting an anti-inflammatory effect [32,34].

The effects of STZ-induced DM and MEL treatment on glial cells have also been evaluated. Baydas et al. [29] investigated the effect of MEL (10 mg/kg, i.p.) on glial reactivity, which is a common feature of brain damage in the hippocampus, cortex, and cerebellum of diabetic Wistar rats and can be triggered by a series of stimuli, such as oxidative stress. DM was shown to cause an increase in astrocyte function markers, glial fibrillary acidic protein (GFAP), and S100B, observed in glial reactivity, while MEL reduced these levels in the three regions analyzed, possibly by enhancing the antioxidant defense system of these glial cells. Wang et al. [35] evaluated changes in astrocytes maintained in cultures with a high concentration of glucose. They reported an increase in the gene expression of several cytokines (TNF-α, IL-6, IL-1β, IL-4, and VEGF) and reactive oxygen species (ROS), and the respective blocking of this increase by MEL (100 nM).

Metwally et al. [33] investigated the effect of MEL (50 mg/kg, i.p.) on central neuropathy caused by STZ-induced DM in Wistar rats. In cerebral neuropathy, there was neurodegeneration: myelinopathy, axonopathy, microglial, and astrocytic activation and vascular damage. Treatment with MEL did not alter the state of hyperglycemia, but it did improve oxidative stress and neurodegeneration.

Tiong et al. [36] when analyzing the effects of a high concentration of glucose in cultured Schwann cells, reported that apoptosis was triggered by oxidative stress related to mitochondria and depolarization of the mitochondrial membrane potential. The addition of MEL (0.5–10 µM) to cultured Schwann cells for 24 h resulted in inhibition of ROS generation, restoration of mitochondrial membrane potential, and inhibition of apoptosis.

The effects of STZ-induced DM and its treatment with MEL on neurons and the nervous system have also been evaluated. Negi et al. analyzed the effect of STZ-induced DM and treatment with MEL (3 or 10 mg/kg, p.o.) and nicotinamide (100 or 300 mg/kg, p.o.) on neural functional, biochemical, and behavioral parameters in Sprague-Dawley rats [34,37]. DM reduced motor nerve conduction velocity, sciatic nerve blood flow, latency in the tail flick test (thermal hyperalgesia), and paw withdrawal pressure in the von Frey test (mechanical hyperalgesia), all of which were improved by treatment with MEL alone or in combination with nicotinamide. Hyperglycemia and loss of body weight resulting from DM induction were not altered by MEL treatment. The authors suggested that vascular changes may have led to inadequate nerve nutrition and, consequently, to ATP-dependent pump failure, which may have resulted in changes in RMP and other cell functional parameters [37]. Seyit et al. [38] corroborated previous findings, reporting that MEL (10 mg/kg) increased nerve conduction velocity and amplitude in rats that presented a reduction in these parameters after DM induction. Negi et al. reported that MEL (3–10 mg/kg, p.o.) reduced the DM-induced expression of the NF-κB (nuclear factor-kappa B) cascade of pro-inflammatory cytokines (TNF-α and IL-6), iNOS, and cyclooxygenase-2 levels in the sciatic nerve of Sprague-Dawley rats, resulting in inhibition of the inflammatory cascade [34]. The authors suggested that MEL may modulate neuroinflammation by reducing the activation of the NF-κB cascade and the nuclear erythroid 2-related factor 2 (Nrf2) pathway, which may be responsible, at least in part, for its neuroprotective effect on DN.

In another study, MEL administered orally at 150 mg/kg inhibited the nociceptive behavior induced by formalin; at 300 mg/kg, the allodynic effect of mechanical stimulation of the paw induced by STZ-induced DM in Wistar rats [39]. The authors concluded that MEL could reverse abnormal pain processing mechanisms in DM. Furthermore, it was observed that K-185 (MT_2_ receptor antagonist) blocked, while naltrindole (δ opioid receptor antagonist) and naltrexone (a non-selective opioid receptor antagonist) reduced the antinociceptive effect of MEL. Although the literature suggests that MEL does not bind to opioid receptors, the authors suggested that there may be interactions between MEL and opioid peptides [39].

Jangra et al. [31] performed behavioral tests to investigate the effect of MEL (3–10 mg/kg, p.o.) and nicotinamide (300 and 1000 mg/kg, p.o.) on STZ-induced neurochemical and neurobehavioral changes in Sprague-Dawley rats. An open-field behavioral analysis showed that DM increased supported and unsupported rears, while MEL only improved the unsupported rears, the combination of MEL and nicotinamide improved supported and unsupported rears. Moreover, DM reduced rotarod performance and the avoidance response test, while MEL improved both [31]. Regarding neurochemical tests, DM increased the MDA and Gamma-Aminobutyric acid levels and reduced Nicotinamide Adenine Dinucleotide, acetylcholinesterase, and glutamate levels in the hippocampus. MEL also reversed all these changes; however, about glutamate levels, the reversal occurred only when MEL was combined with nicotinamide [31]. Zhang et al. [40] reported that treatment with MEL (10 mg/kg, i.p.) had a beneficial therapeutic effect on changes in erectile function and neuropathy of the penile dorsal nerve and major pelvic neural ganglia induced by DM in Sprague-Dawley rats. In addition, there was an increase in collagen deposition, oxidative stress, and increased levels of phosphorylated p-38 protein in the penis, which were reduced by MEL (10 mg/kg, i.p.) [40]. The authors stated that this protein is involved in a series of diabetic complications, such as peripheral neuropathy.

Gurel-Gokmen et al. [30] reported that MEL (10 mg/kg, i.p.) may be effective against neuropathy, as, through histological observations, MEL was found to reduce neuronal degeneration in the cortex when compared to the untreated diabetic group. Finally, Maher et al. [32] investigated the effect of MEL (10 mg/kg, i.p.) on neuroinflammation caused by T2DM induced by a high-fat diet in Sprague-Dawley rats. In the DM group, hyperglycemia was associated with increased levels of pro-inflammatory cytokines (TNF-α and IL-6), MDA, and leptin. In the group treated with MEL, a reduction in hyperglycemia, pro-inflammatory cytokines, and leptin, and increased levels of adiponectin, an anti-inflammatory adipokine, were observed. In addition, MEL improved cerebral oxidative stress and increased TAC and GSH levels, while MDA levels were reduced.

Che et al. [41] evaluated the possible neuroprotective effect of MEL (10 mg/kg, p.o.) in DM-induced neuronal death. According to the authors, MEL reduced neuronal death in mice and inhibited neuronal pyroptosis and excessive autophagy. Magar et al., [42] in turn, investigated the possible relationship between mitochondrial dysfunction and DN. DM was induced in rats by injecting STZ (45 mg/kg) and ingesting fructose (10%) in drinking water for 8 weeks. The model triggered thermal allodynia, reduced nerve conduction velocity, and led to moderate neuronal degeneration in the sciatic nerve. Treatment with MEL (25 and 50 mg/kg, p.o.) reversed these effects when applied alone or in combination with gabapentin (100 mg/kg, p.o.). MEL treatment also increased the hepatic gene expression of peroxisome proliferator-activated receptor-gamma coactivator 1-α and Mitochondrial transcription factor A, suggesting an increase in mitochondrial biogenesis.

**Figure 2:**
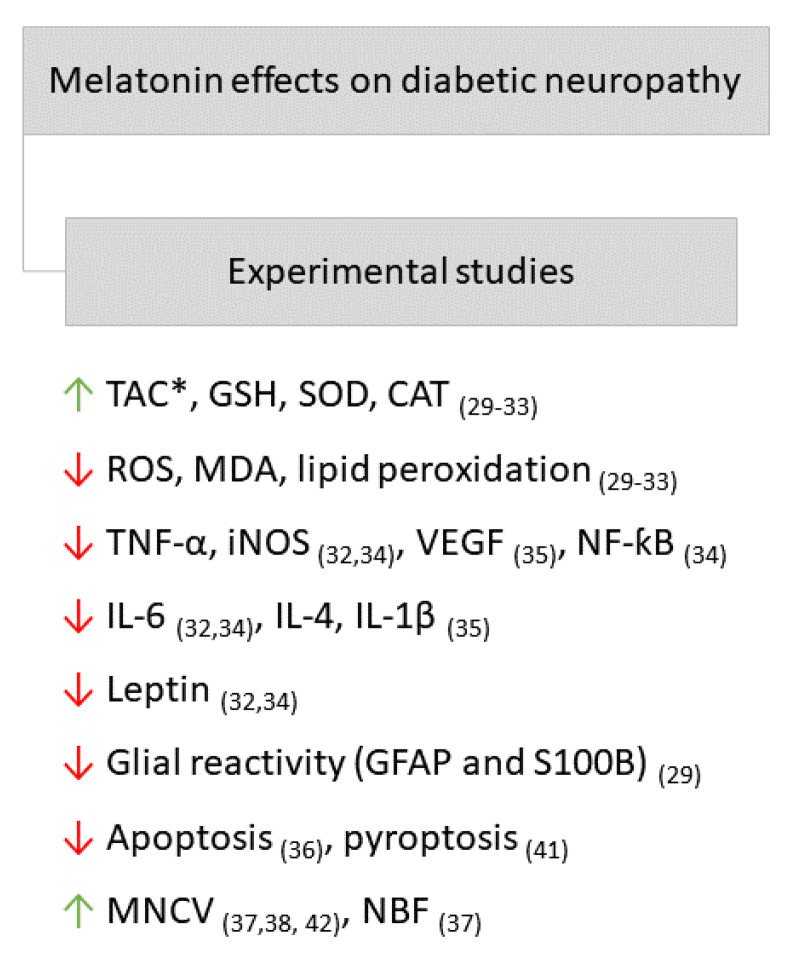
Main effects of MEL on diabetic neuropathy in experimental studies. Arrows point down: decrease; arrows point up: increase. *, TAC: total antioxidant capacity; GSH: glutathione-S-transferase; SOD: superoxide dismutase; CAT: catalase; ROS: reactive oxygen species; MDA: malondialdehyde; TNF-α: Tumor Necrosis Factor alpha; iNOS: inducible nitric oxide synthase; VEGF: vascular endothelial growth factor; NF-κB: nuclear factor-kappa B; IL-6: interleukin 6; IL-4: interleukin 4; IL-1β: interleukin 1 beta; GFAP: glial fibrillary acidic protein; MNCV: motor nerve conduction velocity; NBF: nerve blood flow. The respective references can be found subscribed in parentheses.

As shown in the studies cited above, MEL acts through different mechanisms, including by mediating antioxidant, anti-inflammatory, and antinociceptive effects. Thus, in general, MEL exerted protection over the central and peripheral nervous systems and glial cells in studies using experimental animals. The main MEL effects on DN in experimental studies can be found in Figure 2.

#### 2.2.3. Effect of Melatonin Treatment on Diabetic Neuropathy in Clinical Studies

Shokri et al. [43] conducted a randomized, double-blind clinical trial to assess the efficacy of MEL as an adjuvant to pregabalin (150 mg/day) for pain relief in patients with painful diabetic neuropathy. Subjects undergoing this trial received MEL for 1 week at a dose of 3 mg/day, followed by a dose of 6 mg/d for 7 weeks. These MEL-treated patients reported a considerable reduction in pain and pain-related sleep interference scores when compared to the control group.

In contrast to therapeutic replacement of MEL in age-related hypomelatoninemia, in which case the aim is to achieve physiological plasma concentration, there is great uncertainty regarding the therapeutic doses in pathological conditions, as can be seen in the works cited above. There is neither standardization regarding the tested doses of MEL nor available knowledge that clearly informs a dose or a narrow range of doses for the treatment of DN. Furthermore, most studies on MEL and DN were performed using experimental animals, which makes it even more difficult to propose an effective dose and route of administration of MEL for the treatment of DN. However, some general guidelines should be followed as suggested for the therapeutic use of MEL in metabolic diseases [44].

#### 2.2.4. Effect of Melatonin Treatment on Diabetic Retinopathy in Experimental Studies

Regarding the effects of MEL on animal models of DR, different approaches have been used. Buonfiglio et al. [45] analyzed the diurnal profile of the MEL content of the retina and the regulation of its synthesis in the retinas of Wistar rats with STZ-induced DM. Diabetic animals showed a reduction in MEL daily rhythm, corroborating the findings in humans, and the activity of arylalkylamine N-acetyltransferase (AANAT) in the retina. These effects were reversed by insulin treatment. No necrosis or apoptosis was observed in the retinal cells of the STZ-induced DM group compared to the control group.

Several authors aimed to characterize the effect of MEL treatment on animals with DM and with the development of DR through different types of analyses. One of the most common analyses was on oxidative stress, which was increased by DM and reversed by MEL. It has been reported that DM causes increased levels of nitrotyrosine, MDA, and lipid peroxidation, and reduced levels of GSH [46,47,48]. DM also causes increased release of pro-inflammatory cytokines, such as TNF-α, IL-1β, iNOS, and IL-6, while MEL exerts anti-inflammatory effects by reducing these markers [46,49,50].

Salido et al. [51] analyzed the effect of MEL on retinal changes in Wistar rats with STZ-induced DM. Treatment with MEL (20 mg) was performed through subcutaneous pellet implantation. The DM model resulted in the development of hyperglycemia, reduction of a and b waves and oscillatory potential amplitude of electroretinography (ERG), and an increase in GFAP and vascular endothelial growth factor (VEGF) levels. Data from previous studies corroborate the alterations in a and b waves of the ERG by DM [52,53]. MEL did not affect glucose metabolism, but did prevent a decrease in the a wave, b wave [51,53] and the amplitude of the oscillatory potential of the ERG of diabetic rats. In addition, MEL prevented the increase in GFAP levels in Müller cells and VEGF in the retina. MEL also prevented the decrease in retinal catalase activity [51].

Li et al. [54] reported a neuroprotective effect of MEL (10 mg/kg, p.o.) on the retinal neurons of diabetic rats. The treatment caused a reduction in the rate of apoptosis of retinal cells, reduced caspase-3 mRNA expression, and increased mRNA expression and Mn SOD and Cu-ZN SOD activity, exerting a beneficial effect on retinal neuronal apoptosis.

Xie et al. [55] evaluated the possible protective effects of MEL (100 µM) and interleukin 4 (IL-4) (40 ng/mL) on inflammatory markers involved in DR. In vitro culture of human retinal endothelial cells (RECs) and retinal pigment epithelial cells (RPE), which consist of the inner retinal barrier and the outer barrier, respectively, in a medium containing a high concentration of glucose or IL-1β. IL-4 and MEL inhibited the expression of several cytokines, including VEGF, intercellular adhesion molecule-1, matrix metalloproteinase 2 (MMP2), and MMP9, induced by high glucose and IL-1β in human RECs and RPE cells. These results suggest that these immunoregulatory factors may protect the retina due to their anti-inflammatory effects. Jiang et al. [56] also investigated whether MEL protected cultured Muller cells from oxidative damage caused by a high concentration of glucose (30 mM). Glucose has been reported to trigger an increase in the expression of MT_1_ and MT_2_ receptors, as well as an increase in the production of VEGF, which regulates retinal vascular proliferation and leakage. Exposure of Muller cells to MEL (10 nM–0.1 mM) resulted in reduced VEGF production, suggesting that MEL can trigger a protective effect in the retina during DR.

Jiang et al. [46] evaluated the effect of MEL on retinal injury in Sprague-Dawley rats and its mechanisms. Depletion of GSH levels and downregulation of glutamate cysteine ligase (GCL), the step-limiting enzyme of GSH, were observed in the retinas of diabetic rats. In addition, there was an increase in the levels of the inflammatory cytokines TNF-α, IL-1β, and iNOS, and an increase in the expression of MT_1_ and MT_2_ receptors. MEL (10 mg/kg, i.p.), in turn, significantly increased GCL levels, retained Nrf2 in the nucleus, and stimulated Akt phosphorylation. The production of pro-inflammatory cytokines and proteins, including IL-1β, TNF-α, and iNOS, was inhibited by MEL through the NF-κB pathway. In addition, MEL prevented a significant decrease in the a- and b-wave amplitudes on ERG corroborating the findings of a previous study [51]. Thus, the authors suggested that MEL modulation in the retina and its receptors may represent a potential intervention strategy for DR.

Tu et al. [50] evaluated the pharmacological effect of MEL on the activation of Müller cells, the secretion of proinflammatory cytokines, and the regulatory function of MEG3, a long non-coding RNA. For this purpose, male C57BL/6 mice with DM induced by STZ and a high-fat diet were treated with MEL (10 mg/kg, i.p.) 6 weeks after induction for 7 consecutive days. MEL inhibited gliosis activation and the production of inflammatory cytokines (VEGF, TNF-α, IL-1β, and IL-6) in Müller cells, the main retinal glial cells, in in vitro and in vivo DR models. This effect was blocked by luzindole, suggesting the involvement of MEL membrane receptors. The authors suggested that the protective effect of MEL on Müller cells was mediated by MEG3 expression. In 2021, Tu et al. [57] re-evaluated the effects on Müller cells when MEL was administered with an inhibitor of the silent information regulator factor 2-related enzyme (Sirt1), EX-527. It was reported that MEL exerted an antioxidant and anti-inflammatory effect on Müller cells, which was neutralized by EX-527, suggesting that it acts by activating the Sirt1 pathway to protect the retina from damage caused by DM. Ferreira de Melo et al. [49] documented that MEL (10 mg/kg, s.c.) reduced the expression of proinflammatory cytokines (IL-6 and TNF-α), VEGF, and the apoptosis index in retinopathy in Wistar rats with STZ-induced DM. Thus, the authors concluded that MEL supplementation had a therapeutically positive effect by decreasing the synthesis of proinflammatory cytokines, angiogenic factors, and apoptosis in the retina of diabetic rats, suggesting that it is a potential therapeutic resource for the treatment of DR.

Some studies have evaluated the effects of DM on retinal vascular function. Özdemir et al. [48] evaluated the effects of MEL on oxidative stress and vascular damage in Wistar rats with STZ-induced DM. Hyperglycemia caused an increase in nitrotyrosine and MDA levels, suggesting an increase in retinal oxidation. Furthermore, an increase in the levels of angiogenesis modulating factors was observed, such as hypoxia-inducible factor 1α (HIF-1α), VEGF-A, and pigment epithelial-derived factor due to DM. Additionally, the retinas of diabetic rats showed abnormal vascular changes, such as dilation and deformation. Treatment with MEL (10 mg/kg, i.p.), in turn, reduced the levels of nitrotyrosine, MDA, vasomodulating cytokines, and normalized vascular changes in the retina. Thus, the authors suggested that MEL has a potential beneficial effect on retinopathy in diabetic rats due to its antioxidant effect [48].

Mehrzadi et al. [47], using fluorescein angiography, evaluated the effects of MEL treatment on retinal injury in STZ-induced DM Wistar rats. Development of retinal changes in DM rats was observed, which was reversed by treatment with MEL (20 mg/kg, p.o.). Alterations in oxidative stress were also observed; DM caused increased levels of ROS and MDA, both of which were reversed by MEL treatment. The authors concluded that MEL had a protective effect on the retinas of DM-induced rats [47].

Yan et al. [58] investigated the role of MEL in retinal angiogenesis and the internal blood-retinal barrier under high glucose concentrations. Human retinal microvascular endothelial cells (HRMECs) were maintained in a medium with a high concentration of glucose (30 mmol) and exposed to MEL (100 µM) for 24 h. The high concentration of glucose caused increased protein levels of MMP2, MMP9, and VEGF, all of which are associated with angiogenesis in the retina. MEL treatment reduced the expression of these proteins, as well as reduced autophagic dysfunction and inflammatory responses. The high concentration of glucose also suppressed autophagy in HRMECs and increased the expression of IL-1β and TNFα.

Doğanlar et al. [59] used ARPE-19 cultured cells (human retinal pigment epithelium cells) exposed to glucose (25 mM) and hypoxia, induced by deferoxamine mesylate salt, to investigate the integrity of the blood-retinal barrier (BRB) and the potential protective efficacy of MEL on diabetic macular edema, the leading cause of blindness in DR. The model induced hyperpermeability of the BRB and increased expression of genes linked to hypoxia and angiogenesis (HIF1-α and HIF1-β), all of which were reversed by MEL (0.5–1 mM). Tang et al. [53] also found an increase in leakage of the retinal blood vessels in the BRB due to hyperglycemia, while treatment with intravitreal MEL (30 µg) maintained the integrity of the barrier. Glucose also altered the expression of genes involved in mitochondrial function. The expression of fission-related genes (DRP1, hFiS1, MIEF2, and Mff) and mitochondrial fusion (OPA1) were increased by glucose, all of which were prevented by MEL [59]. Glucose also increased the levels of genes related to mitophagy (PINK, BNip3, and NIX), while MEL-treated groups showed lower expression. Based on the data presented, the authors suggested that mitochondrial dysfunction may be involved in the pathology of diabetic macular edema and that MEL may have a therapeutic effect due to its effect on mitochondria [59].

Chang et al. [52] also evaluated the relationship between mitochondria and DR through cells characterized as a 661 W cone-photoreceptor cell line in culture. When exposed to high concentrations of glucose, there was an increase in the gene expression of dynamin-related protein 1 (DRP1), and a reduction in mitofusin 2 and mitochondrial calcium uniporter (MCU). These changes suggest that blood glucose induces mitochondrial fission but reduces the mitochondrial calcium pool and fusion. Treatment with MEL (100 µM) prevented the increase in DRP1 and the decrease in MCU. In the data obtained from diabetic mice, there was a delay in the response of the retina to light, an increase in the vascular area of the eye, and an increase in the mean length of the vessels, suggesting an increase in vascular permeability. Treatment with MEL (10 mg/kg, p.o.) for 3 months reduced the increase in vascular area and mean vessel length, suggesting a protective effect on the actions of DM in the retina.

Unlike the studies cited thus far, Djordjevic et al. [60] evaluated the potential benefit of MEL in the retinas of animals with prediabetes (fasting glucose: 6.51 ± 0.34 mmol/l). Induction occurred by injecting STZ (45 mg/kg, i.p.) and nicotinamide (110 mg/kg, i.p.). The experimental model showed reduced MEL levels and increased levels of pro-angiogenic molecules (VEGF and MMP9). Supplementation with MEL (85 µg/animal/day, p.o.) resulted in increased plasma levels of MEL and reduced levels of VEGF and MMP9. Thus, the authors suggested that MEL may be considered for the initial treatment or prevention of retinal changes associated with prediabetes.

**Figure 3:**
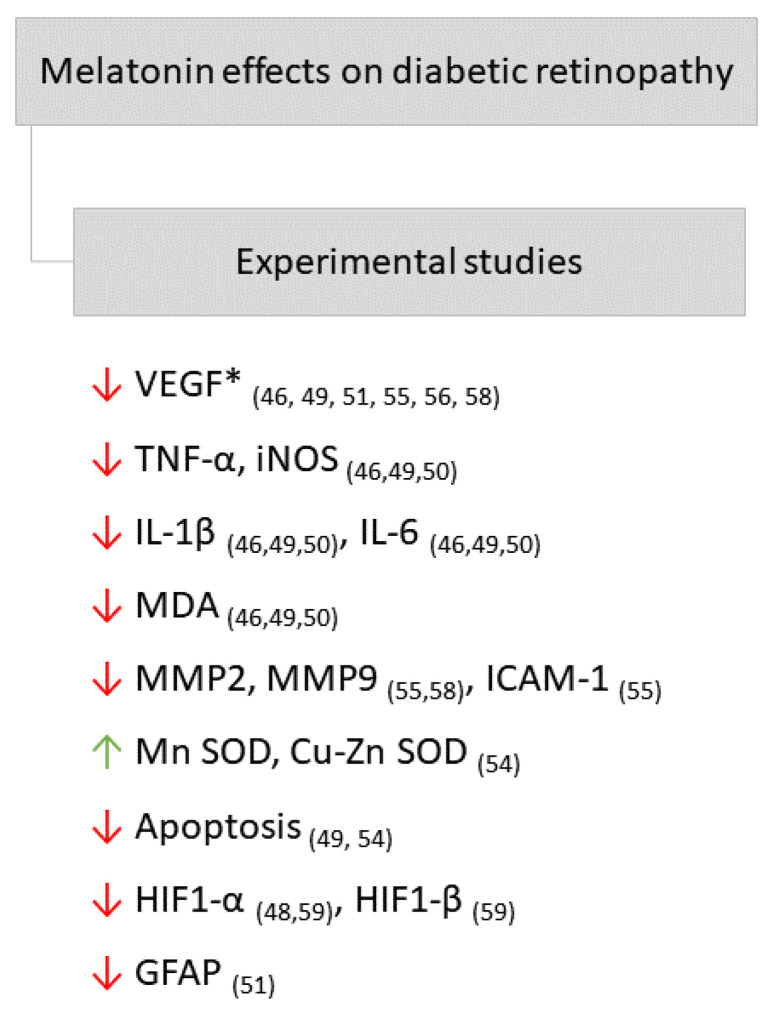
Main effects of melatonin on diabetic retinopathy in experimental studies. Arrows point down: decrease; arrows point up: increase. *, VEGF: vascular endothelial growth factor; TNF-α: Tumor Necrosis Factor alpha; iNOS: inducible nitric oxide synthase; IL-1β: interleukin 1 beta; IL-6: interleukin 6; MDA: malondialdehyde; MMP2: matrix metalloproteinase 2; MMP9: matrix metalloproteinase 9; ICAM-1: intercellular adhesion molecule; Mn SOD: manganese superoxide dismutase; Cu-Zn SOD: Copper zinc superoxide dismutase; HIF1-α: hypoxia-inducible factor alfa; HIF1-β: hypoxia-inducible factor beta; GFAP: glial fibrillary acidic protein. The respective references can be found subscribed in parentheses.

Studies that evaluated the effect of MEL on experimental models of DR suggest that this hormone may exert a protective effect on the retina of diabetic animals through mechanisms similar to those reported in animals with DN, such as antioxidant and anti-inflammatory effects. Furthermore, MEL had an inhibitory effect on apoptosis and improved mitochondrial dysfunction caused by DR. The main effects of MEL on DR in experimental studies can be found in Figure 3.

#### 2.2.5. Relationship of MEL with Diabetic Retinopathy in Clinical Studies and Its Relevance as a Possible Therapeutic Agent

Among the investigations of the relationship between DR and MEL in humans, Hikichi et al. [61] addressed the relationship between the dynamics and magnitude of plasma secretion of this hormone, and DR in patients with and without T2DM. The diabetes group was subdivided into patients with proliferative retinopathy (PDR) and non-proliferative retinopathy (NPDR). MEL secretion has been reported to be lower in the diabetes groups. In patients with diabetes, lower MEL secretion was observed in patients with PDR than in patients with NPDR and non-diabetes patients. A hypothesis suggested by the authors is that the reduction in MEL secretion may have been triggered by autonomic neuropathy. Ba-Ali et al. [62] evaluated the levels of melatonin, cortisol, activity/rest, and sleep quality in patients with and without non-proliferative DR. A significant reduction in mean nocturnal MEL levels and peak daily secretion has been reported in patients with diabetes, regardless of the DR stage, compared to healthy patients. In addition, patients with DR showed an increase in intraday variation in the activity/rest ratio, suggesting a circadian rupture in this population. The authors suggested that circadian disruption is associated with glucose intolerance, obesity, and DM.

Chen et al. [63] evaluated the levels of the urinary MEL metabolite, 6-sulfatoxymelatonin (aMT6s), a reliable marker of MEL production [13], in patients with T2DM without retinopathy, and with PDR or with NPDR. There was a significant reduction in urinary aMT6s levels in patients with PDR when compared to those with NPDR and T2DM without DR. The authors suggested that MEL deficiency may be associated with the pathogenesis of PDR.

Aydin and Sahin [64] evaluated the MEL levels in the aqueous humor of patients with T2DM. It was reported that the concentration of MEL was significantly higher in the aqueous humor of patients with PDR, but not in patients with NPDR, when compared to control non-diabetic patients. A reduction in plasma MEL levels was also reported in the PDR group, but the difference was not statistically significant. The authors suggested that the high levels of MEL in the aqueous humor may be related to the severity of DR.

Reutrakul et al. [65] observed a positive association between the presence and severity of obstructive sleep apnea (OSA) and the presence of DR, with lower nocturnal excretion of aMT6s, signaling this association with lower nocturnal secretion of MEL. Possible relationships between OSA, MEL levels, and glycemic control in patients with T2DM were also explored because, according to the literature, reduced MEL levels and OSA are risk factors for T2DM. An association was observed between the presence and severity of OSA and the presence of DR, with a lower nocturnal secretion of aMT6s.

Sirisreetreerux et al. [66] reported that patients with T2DM and DR had poor sleep quality, greater variability in sleep duration, and low nocturnal production of aMT6s in relation to healthy patients (control group). The authors suggested that the low nocturnal production of aMT6s likely contributed to the development of sleep irregularities, possibly due to poor circadian signaling.

The report published by Wan et al. [67] is the most recent involving human and DR studies. This study aimed to determine the effect of MEL on the risk of developing DR. As the plasma concentration of MEL was reduced in patients with DR, the authors proposed that MEL can be used as a sensitive and specific biomarker for the diagnosis of DR.

## 3. Conclusions

Based on the studies presented, which include many aspects of the pathophysiology of diabetic degeneration, such as oxidative stress, neuroinflammation, neuropathic pain, and glial reactivity, there are clear indications that MEL has a beneficial effect on DN and DR and is therefore promising from a therapeutic point of view. In addition, among other aspects worth mentioning, some effects of MEL are antagonistic to those triggered by DM. In situations in which DM, a progressive degenerative disease, causes RMP depolarization, increased excitability, and oxidative stress in some neural tissues, as well as decreased Na^+^K^+^ATPase activity in neuronal tissues, MEL causes opposite changes, strengthening the possibility of its use in the treatment of DM and its complications.

However, there is a certain difficulty in developing a unified view, as the studies present different approaches with variations in the administration route, DM induction model, characterization time of the disease and its complications, and the period and dose of MEL treatment. Furthermore, the works conducted in the nervous system analyze very different structures without due attention to the temporal course. As DM is characterized as a chronic progressive degenerative disease, monitoring the temporal course is essential.

Finally, most studies do not elucidate the mechanisms of action of MEL as they do not include intrinsic variables of neuronal functioning, such as ionic currents and ion channels related to excitability, conductivity, and synaptic transmission. Thus, according to the studies revised, MEL may be suggested as a promising molecule in the treatment of complications from diabetes mellitus, specifically, DN and DR. However, more studies are needed to establish some key aspects for the treatment, such as dosage, route, and time of administration, as well as the respective mechanisms of action.
